# *Eisenia bicyclis* Extract Repairs UVB-Induced Skin Photoaging In Vitro and In Vivo: Photoprotective Effects

**DOI:** 10.3390/md19120693

**Published:** 2021-12-03

**Authors:** Se-In Choi, Hee-Soo Han, Jae-Min Kim, Geonha Park, Young-Pyo Jang, Yu-Kyong Shin, Hye-Shin Ahn, Sun-Hee Lee, Kyung-Tae Lee

**Affiliations:** 1 Department of Pharmaceutical Biochemistry, College of Pharmacy, Kyung Hee University, Seoul 02447, Korea; sein95@khu.ac.kr (S.-I.C.); heesu3620@khu.ac.kr (H.-S.H.); 2014102197@khu.ac.kr (J.-M.K.); 2 Department of Biomedical and Pharmaceutical Science, Graduate School, Kyung Hee University, Seoul 02447, Korea; 3 Department of Life and Nanopharmaceutical Sciences, Graduate School, Kyung Hee University, Seoul 02447, Korea; ginapark0326@khu.ac.kr (G.P.); ypjang@khu.ac.kr (Y.-P.J.); 4 Department of Oriental Pharmaceutical Science, College of Pharmacy, Kyung Hee University, Seoul 02447, Korea; 5 Department of New Material Development, COSMAXBIO, Seongnam 13486, Korea; ykshin@cosmax.com (Y.-K.S.); hsahn@cosmax.com (H.-S.A.); bt-shlee@cosmax.com (S.-H.L.)

**Keywords:** *Eisenia bicyclis*, UVB, photoaging, MMP-1, collagen, MAPK, AP-1, Smad

## Abstract

Chronic exposure to ultraviolet B (UVB) is a major cause of skin aging. The aim of the present study was to determine the photoprotective effect of a 30% ethanol extract of *Eisenia bicyclis* (Kjellman) Setchell (EEB) against UVB-induced skin aging. By treating human dermal fibroblasts (Hs68) with EEB after UVB irradiation, we found that EEB had a cytoprotective effect. EEB treatment significantly decreased UVB-induced matrix metalloproteinase-1 (MMP-1) production by suppressing the activation of mitogen-activated protein kinase (MAPK)/activator protein 1 (AP-1) signaling and enhancing the protein expression of tissue inhibitors of metalloproteinases (TIMPs). EEB was also found to recover the UVB-induced degradation of pro-collagen by upregulating Smad signaling. Moreover, EEB increased the mRNA expression of filaggrin, involucrin, and loricrin in UVB-irradiated human epidermal keratinocytes (HaCaT). EEB decreased UVB-induced reactive oxygen species (ROS) generation by upregulating glutathione peroxidase 1 (GPx1) and heme oxygenase-1 (HO-1) expression via nuclear factor erythroid-2-related factor 2 (Nrf2) activation in Hs68 cells. In a UVB-induced HR-1 hairless mouse model, the oral administration of EEB mitigated photoaging lesions including wrinkle formation, skin thickness, and skin dryness by downregulating MMP-1 production and upregulating the expression of pro-collagen type I alpha 1 chain (pro-COL1A1). Collectively, our findings revealed that EEB prevents UVB-induced skin damage by regulating MMP-1 and pro-collagen type I production through MAPK/AP-1 and Smad pathways.

## 1. Introduction

The effects of sunlight on the skin are profound and are thought to account for up to 90% of visible skin aging [1]. Periodic and continuous exposure to ultraviolet (UV) radiation is a classical and critical factor that contributes to skin aging, known as photoaging. Photoaging is characterized by wrinkles, inflammation, pigmentation, sagging, and dryness. Although UVB accounts for only a small portion of the total UV radiation, it is the most active at damaging the epidermis and dermis of the skin [2].

Several studies have reported that UVB irradiation increases intracellular reactive oxygen species (ROS), such as superoxide anions, hydroxyl free radicals, and hydrogen peroxide [3]. ROS stimulate various signaling pathways and initiate biological processes, including cell death, cellular senescence, and inflammation [4]. ROS directly phosphorylate mitogen-activated protein kinases (MAPKs) and subsequently upregulate activator protein 1 (AP-1), a transcription factor that accelerates matrix metalloproteinase (MMP) expression [5]. MMPs are involved in the degradation of collagen type I, a major extracellular matrix (ECM) component that provides structural support to the skin, cause decomposition of the dermis, and finally leads to skin aging [6]. In contrast, tissue inhibitors of metalloproteinases (TIMPs) inhibit collagen degradation by suppressing MMP function. The transforming growth factor-β (TGF-β)/Smad pathway regulates collagen synthesis against collagen breakdown in the skin [7]. TGF-β binds to its specific cell surface receptor and activates Smad2/3 transcription factors. Phosphorylated Smad2/3, in association with Smad4, is translocated to the nucleus, which subsequently induces the transcription of TGF-β-responsive genes [8]. Therefore, the regulation of the MAPK/AP-1 pathway and TIMP expression related to MMPs and the Smad-related pro-collagen synthesis pathway play an important role in the maintenance of collagen content in the skin. Therefore, these pathways are the main targets of skin photoaging response induced by UVB irradiation.

Nuclear factor erythroid 2-related factor 2 (Nrf2) is a master transcription factor that binds to antioxidant response elements (ARE) and regulates the expression of antioxidant genes, such as NAD(P)H quinone oxidoreductase 1 (NQO1), heme oxygenase-1 (HO-1), and γ-glutamylcysteine synthetase (γ-GCS) [9]. These antioxidant enzymes may protect dermal cells from UV-induced oxidative stress [7].

Many plant extracts have been reported to exhibit anti-photoaging effects based on their anti-oxidative activities and low toxicity, such as *Hydrangea serrata* [10], *Pradosia mutisii* [11], *Artemisia asiatica* [12], soybean [13], sea buckthorn [14], turmeric [15], and pomegranate fruit [16]. Although the topical and oral applications of these plant extracts have been evaluated, a few studies have assessed UV-induced skin damage and the anti-oxidative effects of edible seaweeds [17,18,19,20].

Marine brown algae accumulate polyphenols composed of phloroglucinol units connected in various ways as phlorotannin [21]. These phlorotannins are abundant in brown algae and have been reported to have a variety of health-beneficial biological activities, including antioxidant [22,23], various enzyme inhibitory [24,25], and radioprotective effect [26]. In addition, previous papers reported several algae possessed anti-photoaging effects against UV [27,28,29]. *Eisenia bicyclis* (Kjellman) Setchell (*E. bicyclis*) is a very common brown alga that belongs to the family Laminariaceae and generally inhabits the middle Pacific coast around Korea, Japan, and China. *E. bicyclis* has been previously revealed to exert anti-inflammatory [30], anti-thrombotic [31], anti-diabetic [32], and neuroprotective effects [33]. However, the anti-photoaging effect of *E. bicyclis* and its molecular mechanisms are yet to be explored. Therefore, as a part of our ongoing screening program to evaluate the anti-photoaging potentials of natural resources, we investigated the molecular mechanism underlying the anti-photoaging properties of 30% ethanol extract of *E. bycyclis* (EEB) on UVB-induced photoaging in human dermal fibroblasts (Hs68), human epidermal keratinocytes (HaCaT), and HR-1 hairless mice.

## 2. Results

### 2.1. Identification of Phlorotannins in EEB by UPLC-PDA-ESI-MS

**Figure 1:**
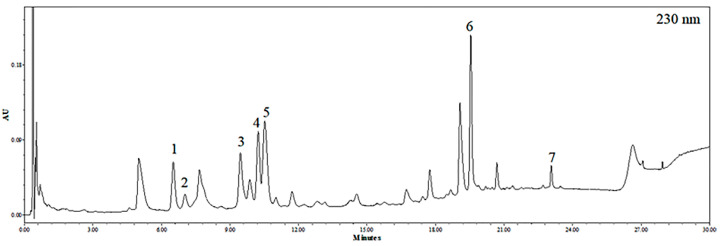
UPLC chromatogram of EEB detected at 230 nm.

The ultra-performance liquid chromatography (UPLC) chromatogram of EEB at 230 nm is shown in Figure 1. By comparing the retention times, mass spectrometric (MS) data, and UV/Vis spectra to those presented in previous literature reports, seven major peaks were identified. The retention time, precursor ion, monoisotopic mass, and UV/Vis λ_max_ of each compound are listed in Table 1. Compound **1** was found to have three phloroglucinol units, with an *m*/*z* value of 373.05449, and was verified to be eckol [34,35]. Compound **2** was confirmed to be phloroeckol owing to an *m*/*z* value of 497.06954, with a mass difference of -2.46 mmu compared to the theoretical value of protonated phloroeckol [35]. Compounds **3–5** were isomers with [M+H]^+^ at *m*/*z* 743.08, and tentatively corresponded to 6,6′-bieckol, 6,8′-bieckol, and 8,8′-bieckol based on a comparison of the chromatographic characteristics with those presented previously [34,36,37]. Compound **6**, observed as [M+H]^+^ at *m*/*z* 743.08, was identified as a dieckol, and is composed of six units of phloroglucinol [35]. Compound **7** was identified as phlorofucofuroeckol A based on its *m*/*z* value [36,38].

### 2.2. EEB Protects against UVB-Reduced Cell Viability in Hs68 Fibroblasts

**Figure 2:**
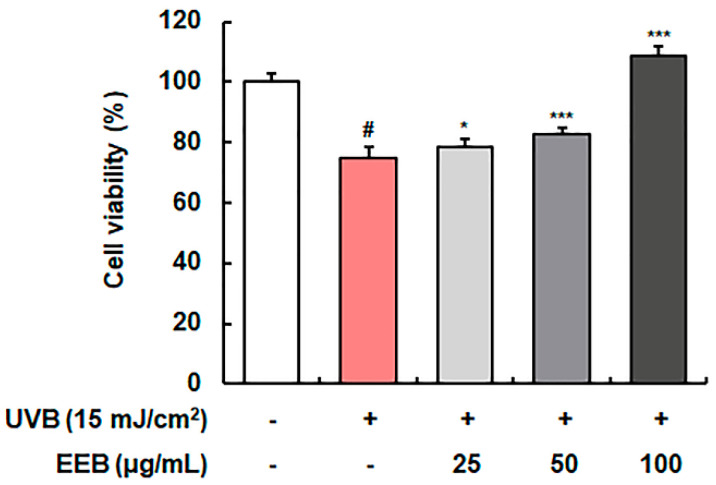
Effect of EEB on UVB-damaged cell protection in Hs68 cells. UVB-irradiated cells were treated with various concentrations of EEB (25, 50, and 100 µg/mL). Cell viability was analyzed using the 3-(4,5-dimethylthiazol-2-yl)-2,5-diphenyl tetrazolium bromide (MTT) assay. Values are represented as mean ± standard deviation (SD). # *p* < 0.05 vs. the non-UVB-irradiated control group; * *p* < 0.05 and *** *p* < 0.001 vs. the UVB-irradiated group.

First, we determined the cytoprotective effects of EEB on UVB-induced Hs68 cells. As shown in Figure 2, UVB-irradiation reduced cell viability to 74.82 ± 3.76% relative to that of control cells (100.00 ± 2.80%); however, cell viability was concentration-dependently and significantly increased by EEB treatment (78.74 ± 2.55%, 82.75 ± 2.26%, and 108.80 ± 3.19% at 25, 50, and 100 µg/mL, respectively). These findings indicate that EEB treatment considerably protects the cells from UVB irradiation-reduced cell viability. Herein, 25, 50, and 100 µg/mL were employed as the effective concentrations of EEB and were thus used in subsequent studies.

### 2.3. EEB Ameliorates UVB-Induced MMP-1 Production and Pro-Collagen Type I Degradation in Hs68 Fibroblasts

**Figure 3:**
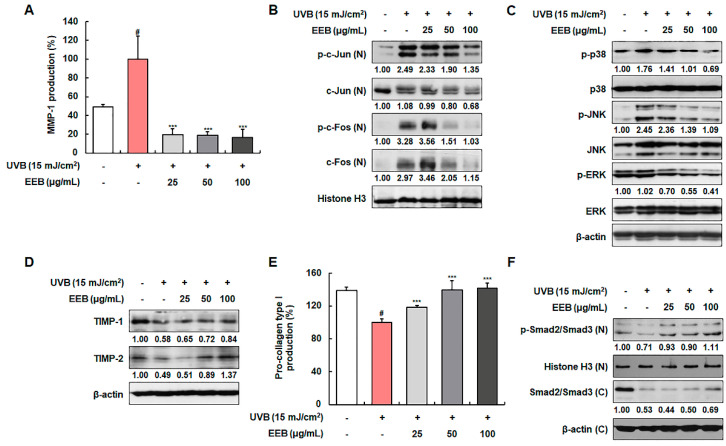
Effects of EEB on the production of MMP-1 and pro-collagen type I, the activation of MAPK/AP-1 and Smad signaling pathways, and TIMPs expression in UVB-irradiated Hs68 fibroblasts. Cells were irradiated with UVB and treated with various concentrations of EEB (25, 50, and 100 µg/mL). (**A**, **E**) The MMP-1 and pro-collagen type I levels in the cell culture media were determined using ELISA kits. Western blot analysis was conducted to determine the protein expression in whole-cell lysates and cytosolic and nuclear fractions. Protein expression of (**B**) p-c-Jun, total c-Jun, p-c-Fos, total c-Fos, (**C**) p-p38, total p38, p-JNK, total JNK, p-ERK, total ERK, (**D**) TIMP-1, TIMP-2, (**F**) p-Smad2/Smad3, and total Smad2/Smad3. Protein levels of AP-1 pathway and p-Smad2/Smad3 normalized to Histone H3, phosphorylated protein levels of MAPK pathway normalized to total form, and protein levels of TIMPs and total Smad2/Smad3 normalized to β-actin were determined using Bio-Rad Quantity One software (Basic; Bio-Rad Laboratories Inc., Hercules, CA, USA). Values are represented as mean ± SD. # *p* < 0.05 vs. the non-UVB-irradiated control group; *** *p* < 0.001 vs. the UVB-irradiated group.

UVB irradiation induces MMP-1 expression, which then degrades collagen fibers [39]. Therefore, we evaluated the inhibitory effects of EEB on MMP-1 production as a known biomarker of skin photoaging. EEB markedly inhibited MMP-1 production by 80.14 ± 5.94%, 81.04 ± 3.61%, and 83.43 ± 8.83% at 25, 50, and 100 µg/mL, respectively, compared with the UVB-irradiated fibroblasts (100.00 ± 24.76%) (Figure 3A). To elucidate the mechanisms involved in the effect of EEB on UVB-induced MMP-1 expression, we examined whether EEB affected the activation of the MAPK/AP-1 pathway and TIMPs expression. As shown in Figure 3B, UVB exposure stimulated the nuclear phosphorylation and expression of AP-1 signaling molecules (c-Jun and c-Fos). However, treatment with EEB strongly suppressed the activation of AP-1. In addition, EEB potently suppressed the UVB-induced phosphorylation of MAPKs (p38, JNK, and ERK), the upstream regulators of AP-1, without affecting the total expression of p38, JNK, and ERK (Figure 3C). EEB also recovered the UVB-reduced expression of TIMP-1 and TIMP-2 in a concentration-dependent manner (Figure 3D). These results indicate that EEB reduces UVB-induced MMP-1 expression by regulating the MAPK/AP-1 signaling pathway and TIMP expression. Next, we determined the effect of EEB on pro-collagen type I production, another biomarker of skin photoaging. The production of pro-collagen type I was reduced by 72.03 ± 3.14% after UVB exposure; however, EEB significantly and concentration-dependently recovered this production (85.01 ± 1.66%, 100.72 ± 7.88%, and 101.76 ± 5.01% at 25, 50, and 100 µg/mL, respectively) (Figure 3E). To explain the mechanisms involved in the effect of EEB on UVB-induced collagen degradation, we examined the Smad pathway in Hs68 cells. As shown in Figure 3F, the phosphorylation and total expression of Smad2/Smad3 were decreased by UVB irradiation, whereas EEB upregulated these reductions in a concentration-dependent manner. These results indicate that EEB recovers UVB-induced collagen degradation by activating the Smad signaling pathway.

### 2.4. EEB Promotes the Skin Moisturization Factors in HaCaT Keratinocytes

**Figure 4:**
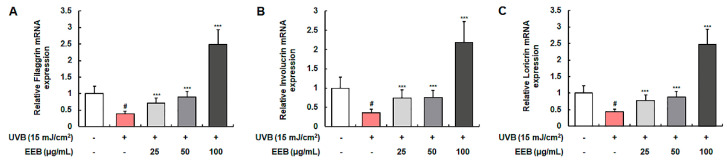
Effects of EEB on skin moisturization in UVB-induced HaCaT keratinocytes. Cells were irradiated with UVB and then treated with various concentrations of EEB (25, 50, and 100 µg/mL). Total cellular RNA was extracted from EEB-treated HaCaT cells. The mRNA levels of (**A**) filaggrin, (**B**) involucrin, and (**C**) loricrin were determined by quantitative real-time RT-PCR (qRT-PCR) and adjusted to GAPDH. Values are represented as mean ± SD. # *p* < 0.05 vs. the non-UVB-irradiated control group; *** *p* < 0.001 vs. the UVB-irradiated group.

Various skin moisturization factors, including filaggrin, involucrin, and loricrin, play a pivotal role in the formation of the epidermal skin barrier and the maintenance of skin hydration [40]. The mRNA levels of filaggrin, involucrin, and loricrin were determined to assess the moisturizing effect of EEB in UVB-induced HaCaT keratinocytes (Figure 4A–C). The mRNA expression of filaggrin, involucrin, and loricrin was markedly reduced by UVB irradiation, but significantly elevated by EEB treatment. EEB did not affect the cell viability of HaCaT at concentrations of 25, 50, and 100 μg/mL (data not shown). These results indicate that EEB improves skin barrier function by restoring natural moisturizing factors.

### 2.5. EEB Reduces UVB-Induced ROS and Enhances Antioxidant Enzymes Expression via the Nrf2 Signaling Pathway in Hs68 Fibroblasts

**Figure 5:**
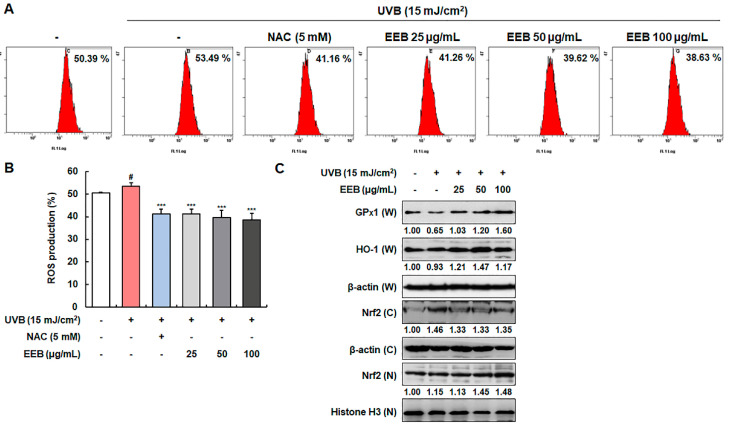
Effects of EEB on ROS production and the antioxidant enzymes expression through Nrf2 signaling in UVB-induced Hs68 cells. Cells were irradiated with UVB and then treated with various concentrations of EEB (25, 50, and 100 µg/mL). (**A**, **B**) Productions of intracellular ROS were stained with H_2_DCFDA and analyzed by flow cytometry. (**C**) Whole-cell lysates and cytosolic and nuclear fractions were analyzed by Western blotting to determine the levels. Protein expression of GPx1, HO-1, and Nrf2. Protein levels of GPx1, HO-1, and cytosolic fraction Nrf2 normalized to β-actin and nucleus fraction Nrf2 normalized to Histone H3 were determined using Bio-Rad Quantity One software (Basic; Bio-Rad Laboratories Inc., Hercules, CA, USA). Values are represented as mean ± SD. # *p* < 0.05 vs. the non-UVB-irradiated control group; *** *p* < 0.001 vs. the UVB-irradiated group.

UVB irradiation is known to induce intracellular ROS production, which causes oxidative damage, promotes the expression of MMPs, and leads to photoaging [41]. As shown in Figure 5A,B, intracellular ROS was produced by UVB irradiation. EEB significantly attenuated UVB-induced ROS production compared to N-acetylcysteine (NAC), a positive control for ROS scavenging. Accordingly, we sought to further determine whether the ROS scavenging activity of EEB was mediated by major antioxidant enzymes, such as glutathione peroxidase 1 (GPx1) and HO-1, which are regulated by the Nrf2 signaling pathway [42]. EEB treatment upregulated GPx1 and HO-1 expression in a concentration-dependent manner (Figure 5C). Moreover, treatment with EEB enhanced the nuclear expression of Nrf2 while simultaneously decreased cytosolic Nrf2 levels. These results indicate that EEB scavenges UVB-induced ROS by enhancing the expression of antioxidant enzymes through the activation of the Nrf2 signaling pathway.

### 2.6. EEB Reduces UVB-Induced Wrinkle Formation in the Dorsal Skin of HR-1 Hairless Mice

**Figure 6:**
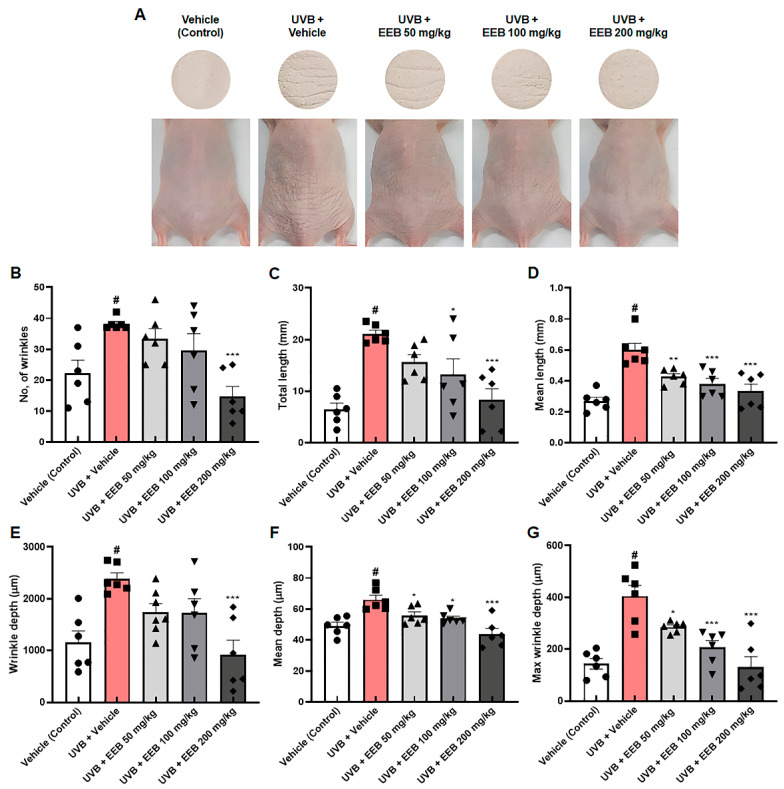
Effects of EEB on wrinkle formation in the dorsal skin of UVB-irradiated HR-1 hairless mice. HR-1 hairless mice were orally administrated with various doses of EEB (50, 100, and 200 mg/kg, p.o.) every 6 days and were irradiated with UVB three times per week for 8 weeks (60 mJ/cm^2^ to 120 mJ/cm^2^). (**A**) Images of the dorsal skin of mice and skin replicas. The replicas were analyzed by Visioline^®^ VL-650. (**B**) Number of wrinkles, (**C**) total length (mm), (**D**) mean length (mm), (**E**) wrinkle depth (µm), (**F**) mean depth (µm), and (**G**) max wrinkle depth (µm) were analyzed. Each symbol in the figures represents an individual mouse of its own group. Values are represented as mean ± standard error of the mean (SEM; *n* = 6). # *p* < 0.05 vs. the vehicle-treated control group; * *p* < 0.05, ** *p* < 0.01, and *** *p* < 0.001 vs. the UVB only-treated group.

We further assessed the effect of EEB on UVB-induced skin aging in vivo. First, we investigated the effect of EEB on wrinkle formation in the dorsal skin of HR-1 hairless mice. Mice were exposed to UVB and orally administrated with vehicle or EEB for 8 weeks. As shown in Figure 6A, the UVB-only treated group had deep and numerous wrinkles in the dorsal skin compared with the vehicle-treated control group; however, oral administration of EEB (50, 100, and 200 mg/kg) markedly reduced wrinkle formation. To quantitatively analyze the degree of wrinkle formation, skin replicas of the dorsal skin of HR-1 hairless mice were obtained at the end of the experiment. The number of wrinkles, total length, mean length, wrinkle depth, mean depth, and maximum wrinkle depth were increased in the dorsal skin of the UVB-only treated group, but significantly mitigated in a dose-dependent manner in the UVB + EEB-treated groups (Figure 6B–G). These results indicate that the oral administration of EEB attenuates UVB-induced wrinkle formation in the dorsal skin of mice.

### 2.7. EEB Inhibits UVB-Induced MMP-1 via the MAPK/AP-1 Signaling Pathway in the Dorsal Skin of HR-1 Hairless Mice

**Figure 7:**
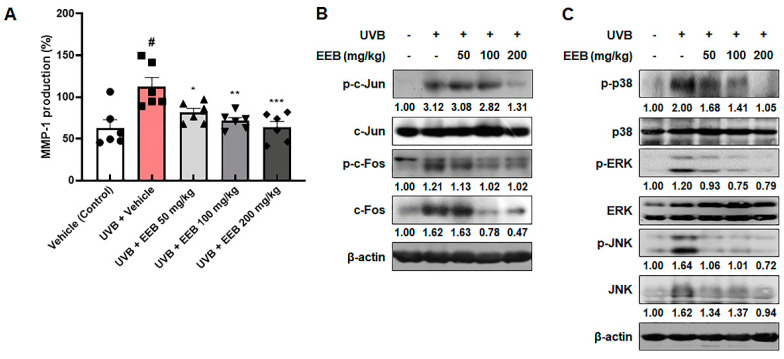
Effects of EEB on MMP-1 production and the MAPK/AP-1 signaling pathway in the dorsal skin of UVB-irradiated HR-1 hairless mice. HR-1 hairless mice were orally administrated various doses of EEB (50, 100, and 200 mg/kg, p.o.) every 6 days and irradiated with UVB three times per week for 8 weeks (60 mJ/cm^2^ to 120 mJ/cm^2^). (**A**) MMP-1 production levels in dorsal skin tissue-lysates were determined with ELISA kits. Each symbol in the figures represents an individual mouse of its own group. Dorsal skin tissue-lysates were analyzed by Western blotting to determine the protein levels. Protein expression of (**B**) p-c-Jun, total c-Jun, p-c-Fos, total c-Fos, (**C**) p-p38, total p38, p-ERK, total ERK, p-JNK, and total JNK. Protein levels of p-c-Jun, p-p38, and p-ERK normalized to total form and p-c-Fos, c-Fos, p-JNK, and JNK normalized to β-actin were determined using Bio-Rad Quantity One software (Basic; Bio-Rad Laboratories Inc., Hercules, CA, USA). Values are represented as mean ± SEM (*n* = 6). # *p* < 0.05 vs. the vehicle-treated control group; * *p* < 0.05, ** *p* < 0.01, and *** *p* < 0.001 vs. the UVB only-treated group.

Following an in vitro study, we investigated whether EEB affects MMP-1 production in UVB-irradiated HR-1 hairless mice. As expected, MMP-1 production was remarkably elevated after UVB irradiation. However, EEB treatment significantly suppressed these increases in a dose-dependent manner (Figure 7A). As shown in Figure 7B, UVB irradiation induced the phosphorylation of c-Jun and c-Fos, which were remarkably inhibited in the UVB + EEB-treated groups. In addition, EEB treatment reduced the UVB-induced phosphorylation of p38, ERK, and JNK (Figure 7C). Interestingly, the inhibitory effects of EEB on the phosphorylation of c-Fos and JNK were regulated via the suppression of their expression. These data indicate that the oral administration of EEB suppresses the MAPK/AP-1 signaling pathway, which consequently reduces UVB-induced MMP-1 synthesis.

### 2.8. EEB Alleviates UVB-Induced Skin Thickening and Collagen Degradation in the Dorsal Skin of HR-1 Hairless Mice

**Figure 8:**
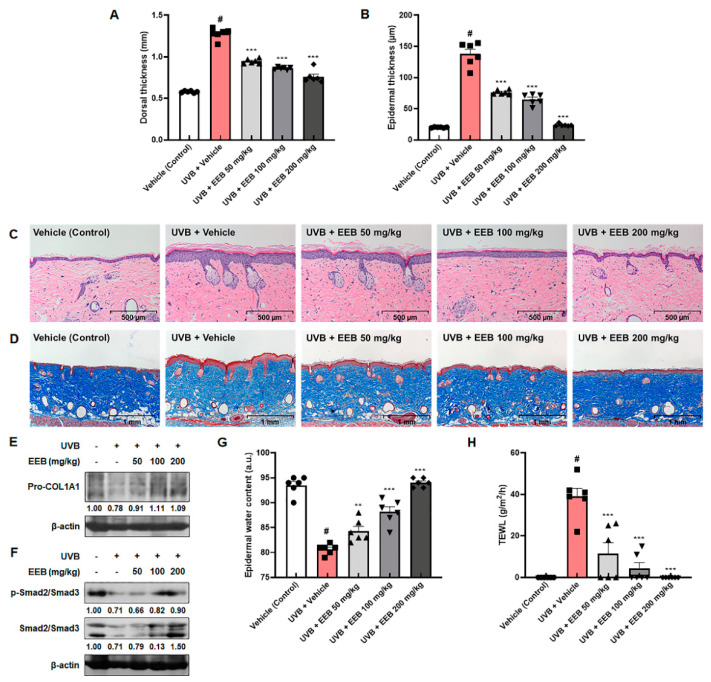
Effects of EEB on skin thickening, collagen degradation, and skin hydration in the dorsal skin of UVB-irradiated HR-1 hairless mice. HR-1 hairless mice were orally administrated with various doses of EEB (50, 100, and 200 mg/kg, p.o.) every 6 days and irradiated with UVB three times per week for 8 weeks (60 mJ/cm^2^ to 120 mJ/cm^2^). (**A**) Dorsal skin thickness was measured using a caliper. (**B**) Histogram of epidermal thickness measured using the (**C**) H&E-stained images of dorsal skin tissues. Scale bar = 500 µm. (**D**) Masson’s trichrome-stained images of dorsal skin tissues. Scale bar = 1 mm. Dorsal skin tissue-lysates were analyzed by Western blotting to determine the protein levels. Protein expression of (**E**) Pro-COL1A1, (**F**) p-Smad2/Smad3, and total Smad2/Smad3. (**G**) The epidermal water content and (**H**) TEWL were measured using the GPSkin Barrier^®^ in the dorsal skin of UVB-irradiated HR-1 hairless mice. Each symbol in the figures represents an individual mouse of its own group. Protein levels of pro-COL1A1 and Smad pathway normalized to β-actin were determined using Bio-Rad Quantity One software (Basic; Bio-Rad Laboratories Inc., Hercules, CA, USA). Values are represented as mean ± SEM (*n* = 6). # *p* < 0.05 vs. the vehicle-treated control group; ** *p* < 0.01 and *** *p* < 0.001 vs. the UVB only-treated group.

Histological examination was conducted to assess the effects of EEB on skin thickening and collagen degradation in the dorsal skin of HR-1 hairless mice. UVB exposure resulted in the swelling and thickening of dorsal skin in HR-1 hairless mice, which appeared to be reversed dose-dependently by UVB + EEB-treated groups (Figure 8A). The hematoxylin and eosin (H&E) stained dorsal skin showed that UVB exposure significantly increased the thickness of the epidermis to 137.62 ± 7.77 µm compared with the vehicle-treated control skin (20.13 ± 0.30 µm), whereas oral administration of EEB significantly suppressed epidermal thickness (79.51 ± 1.51 µm, 71.29 ± 1.20 µm, and 22.83 ± 0.21 µm at 50, 100, and 200 mg/kg, respectively) (Figure 8B,C). The changes in collagen fibers were also observed using Masson’s trichrome staining. Compared to the vehicle-treated control group, the UVB-only treated group showed decreased abundance and density of collagen fibers (Figure 8D). However, UVB-induced damage in collagen fibers was effectively restored in the UVB + EEB-treated groups. As shown in Figure 8E, the expression of pro-collagen type I alpha 1 chain (pro-COL1A1) was enhanced in the UVB + EEB-treated groups compared with the UVB-only treated group. Based on the in vitro results, we evaluated the expression of p-Smad2/Smad3 and total Smad2/Smad3 in UVB-irradiated HR-1 hairless mice. Both the phosphorylation and expression of Smad2/Smad3 were strongly elevated in the UVB + EEB-treated groups (Figure 8F). We proceeded to estimate the moisturizing effect of EEB by measuring the epidermal water content and transepidermal water loss (TEWL) in the dorsal skin surface of HR-1 hairless mice. As shown in Figure 8G,H, decreased epidermal water content and increased TEWL were observed in the UVB-only treated group. However, oral administration of 50, 100, and 200 mg/kg EEB significantly recovered the epidermal water content to 84.33 ± 0.92, 88.17 ± 1.01, and 94.00 ± 0.37, respectively, compared with that of the UVB-only treated group (80.67 ± 0.42). The increased TEWL was also significantly and dose-dependently ameliorated in the vehicle-treated control group. These results suggest that oral administration of EEB reduces skin thickness and exhibits a moisturizing effect by improving the impaired distribution of collagen fibers in the dermis, epidermal water content, and TEWL in the UVB-induced photoaging animal model.

## 3. Discussion

Chronic exposure to UVB promotes skin photoaging, and photodamaged skin exhibits symptoms of inflammation, such as erythema, edema, and pain [43]. UVB potently induces inflammatory molecules in the skin, including inducible nitric oxide synthase (iNOS), cyclooxygenase-2 (COX-2), tumor necrosis factor-alpha (TNF-α), and interleukins [44]. The extract of *E. bicyclis* has previously been reported to have anti-inflammatory properties [30,45]. Suppressing inflammatory responses could serve as an approach to prevent photoaging. We identified seven phlorotannins from EEB, including eckol, phloroeckol, 6,6′-bieckol, 6,8′-bieckol, 8,8′-bieckol, dieckol, and phlorofucofuroeckol A, which possess anti-inflammatory properties and regulates inflammatory mediators and cytokines [30,46,47]. Based on previous reports, the anti-inflammatory activities of EEB and its bioactive compounds can be considered to inhibit UVB-induced skin photoaging. Among these components, eckol and dieckol have been observed as anti-photoaging agents with inhibitory effects on MMP-1 expression [26,48]. Consistently, we found that eckol and dieckol isolated from EEB attenuated UVB-induced MMP-1 production (Figure S1). Moreover, we found the recovery effects of eckol and dieckol on reduced pro-collagen type I production in UVB-irradiated Hs68 cells. Accordingly, we suggest that eckol and dieckol are active components of EEB that exhibit anti-photoaging activity. The molecular mechanisms and in vivo efficacy of eckol and dieckol in UVB-induced skin photoaging need to be further investigated. In previously reported studies, it is known that the diverse extracts of alga exert various improvement effects against UV-induced photodamage. *Corallina pilulifera* inhibits the MMP-2 and MMP-9 expression and free radical oxidation against UV-induced photoaging in vitro [27]. Fermented *Gelidium amansii* and *Cirium japonicum* extract mixture ameliorated UVB-induced ECM remodeling in vitro, and attenuated photodamage via an anti-wrinkle effect and a moisturizing effect in vivo [28]. Another alga, *Polysiphonia morrowii* Harvey, has a photo-protective effect by apoptosis inhibition and antioxidant enzyme induction [29]. However, there are no reports on the anti-photoaging activity of *E. bicyclis*.

We set up the UVB irradiation method used in our study with reference to the previous reports on anti-photoaging effect using UVB-irradiated Hs68 cells and HaCaT cells, and UVB-exposed HR-1 hairless mice [10,49,50,51]. Since the dorsal skin of mice used in the in vivo experiment is a skin tissue containing epidermis and dermis, stronger and more persistent UVB exposure was required than in the in vitro experiment using Hs68 cells and HaCaT cells cultured as a single layer to induce photoaging [10]. For the first time, we evaluated the photoprotective effect of EEB and its underlying mechanisms in this study. UVB-induced MMP-1 production and subsequent degradation of pro-collagen type I are major events in photodamaged skin. EEB was found to potently inhibit MMP-1 production by regulating TIMP expression and the activation of the MAPK/AP-1 signaling pathway. Dieckol has recently been reported to protect against UVB-induced skin damage by regulating the MAPK/AP-1 pathway in human dermal fibroblasts [26]. Thus, dieckol could contribute to the regulatory effect of EEB on MAPK/AP-1 activation. Several phlorotannins in EEB, such as dieckol, 6,6′-bieckol, and 8,8′-bieckol, are known to possess inhibitory effects on nuclear factor-κB (NF-κB), another transcription factor that upregulates MMP expression and inflammatory responses [26,52,53,54]. ERK and p38 of MAPK are also responsible for NF-κB activation in addition to AP-1 via the phosphorylation of mitogen- and stress-activated kinase 1 (MSK1) [55]. Owing to the suppression of the MAPK pathway by EEB containing various phlorotannins, the effect of EEB on NF-κB activation is worthy of further investigation. In this study, EEB restored pro-collagen type I expression by stimulating the Smad signaling pathway. The Smad pathway is initiated by the activation of the TGF-β receptor, which is activated by numerous types of TGF-β superfamily, including TGF-β, activins, inhibins, growth differentiation factors (GDFs), bone morphogenetic proteins (BMPs), and anti-Müllerian hormone (AMH) [56]. TGF-β and activin are activators of Smad2/3, which enhance collagen synthesis [57]. GDF11 is a key growth factor that can proliferate or differentiate skin cells to repair damage [58]. BMPs are involved in the activation of Smad1/5/8 signaling, which plays a crucial role in skin development and homeostasis by modulating cell adhesion, motility, and ECM remodeling [59,60,61]. In terms of the activation of Smad signaling, EEB increased the expression of these TGF-β ligands. Taken together, the combined effects of EEB on both the inhibition of collagen degradation and enhancement of collagen synthesis pathways contribute to the protection against UVB-induced damage.

Edible brown algae and their bioactive compounds have been previously evaluated for their skin care properties via ROS scavenging [62,63,64,65,66]. Intracellular ROS cause oxidative stress and participate in the processes of inflammation, DNA damage, and degradation of tissue structures [67]. In response to the excessive production of ROS, antioxidant enzymes mediated by Nrf2 are produced to manage ROS production [68]. Our results showed that EEB ameliorated UVB-induced intracellular ROS generation in fibroblasts. EEB also enhanced the expression of antioxidant enzymes, such as GPx1 and HO-1 via Nrf2 activation. Thus, EEB can be employed as an antioxidant agent to prevent skin oxidative damage caused by UVB. ROS mediates not only cellular antioxidant systems, but also skin damage and inflammation by stimulating the MAPK pathway [59]. Therefore, ROS might be the main upstream target of EEB in the protection against UVB-induced skin damage.

Skin hydration is a promising target for skin barrier function. Filaggrin is a filaments-aggregating protein that interacts with other proteins and maintains the structural integrity of the stratum corneum. Products released from degraded filaggrin assist in water retention [69]. Involucrin and loricrin are involved in the formation of the cornified envelope, the cohesion of corneocytes, and the consequent enhancement of skin barrier function [70,71]. In this study, we found that EEB recovered the moisturizing and skin barrier proteins, filaggrin, involucrin, and loricrin in UVB-induced damaged keratinocytes. Owing to the moisture gradient between the stratum corneum and the deeper dermal layers, water diffuses and evaporates from the inner layers to the skin surface, which is referred to as TEWL [72]. TEWL can be used as an indicator of the degree of skin hydration and internal barrier properties; a lower TEWL highlights healthy skin conditions with a higher water holding capacity [73]. Oral administration of EEB significantly alleviated the UVB-induced decrease in epidermal water content and increased TEWL. These results demonstrate the skin hydration potential of EEB. Hyaluronic acid (HA) is a widely distributed glycosaminoglycan that is synthesized and degraded by specific enzymes called HA synthases and hyaluronidases. HA binds to water molecules and induces retention to maintain skin moisture [74]. Aquaporin 3, a cell membrane-bound water channel, is directly involved in skin hydration by transporting water and glycerol [75]. Further assessments of the effect of EEB on these additional moisturizing factors and its underlying mechanisms could enhance the moisturizing effect of EEB.

For decades, cosmetic research on skin care has focused on topical applications. However, recent studies have shown that oral supplementation with bioactive products could alleviate skin aging [76,77,78]. The present study also revealed the protective effect of oral administration of EEB and its underlying mechanisms in UVB-induced damaged fibroblasts and keratinocytes. In addition, our in vivo study demonstrated the anti-photoaging effect of EEB, which is demonstrated by the amelioration of UVB-induced wrinkle formation, skin thickness, and skin dehydration. According to the biochemical analyses using mouse plasma, the plasma levels of glutamic oxaloacetic transaminase (GOT), glutamic pyruvic transaminase (GPT), blood urea nitrogen (BUN), and creatinine in the EEB-treated groups were within the normal ranges, indicating the safety of the oral administration of EEB (Figure S2). Based on these data, EEB could be applied to humans as a promising nutraceutical for skin health to improve wrinkled and dried skin.

## 4. Materials and Methods

### 4.1. Preparation and UPLC-PDA-ESI-MS Analysis of EEB

Aerial parts of *E. bicyclis* were collected from Ulleungdo Island, Korea. It was classified by miDNA Genome Research Institute (Gunsan, Korea). A voucher specimen (COS2007) of the species has been deposited at the herbarium of R&I Center, COSMAX BIO, Seongnam, Korea. Washed and dried aerial parts of *E. bicyclis* (281.8 kg) were extracted with 30% ethanol at 60 °C for 5 h followed by evaporation and sterilization afforded a dried extract residue (94.1 kg, 33.4%). The EEB was dissolved in 30% ethanol to set a concentration of 20 mg/mL. The sample solution was loaded into a Sep-pak^®^ C18 cartridge (Waters Corp., Milford, MA, USA) and filtered through a 0.2 µm polyvinylidene fluoride syringe filter (Whatman International Ltd., Maidstone, Kent, UK) before being applied to UPLC analysis. UPLC analysis was performed with a Waters Corp. (Milford, MA, USA) Acquity™ H-Class UPLC system, including a quaternary solvent manager, photodiode array (PDA) detector, cooling auto-sampler, and oven enabling control of the temperature of the analytical column. For MS analysis, a JMS-T100TD (AccuTOF-TLC) (JEOL Ltd., Tokyo, Japan) spectrometer equipped with electrospray ionization (ESI) source was employed. The chromatographic separation was achieved on the Kinetex^®^ 1.7 µm C18 100Å UHPLC column 2.1 × 50 mm (Phenomenex, Torrance, CA, USA), thermostated at 30 °C. The mobile phase consisted of 0.1% formic acid in acetonitrile (solvent A) and 0.1% formic acid in water (solvent B). The gradient condition of the mobile phase was 0–2 min, 8%; 2–15 min, 8% to 15%; 15–25 min, 15% to 30%; 25–27 min, 30% to 100%; 27–30 min, 100%, as percentage of solvent A. The flow rate was 0.3 mL/min, and the injection volume was 2.0 µL. The monitored wavelength of the PDA detector was 230 nm. The conditions of MS analysis in the positive ion mode were as follows: nitrogen gas flow rate, 1.0 L/min (nebulizing gas) and 3.0 L/min (desolvating gas); ring lens voltage, 10 V; orifice 1 voltage, 80 V; orifice 2 voltage, 5 V; scan range, *m/z* 50–1000; detector voltage, 2000 V; peak voltage, 1000 V; bias voltage, 31 V; pusher bias voltage, −0.36 V; desolvating chamber temperature, 250 °C; orifice 1 temperature, 80 °C.

### 4.2. Cell Culture, UVB-Irradiation, and Sample Treatment

Hs68 and HaCaT cells were purchased from the American Type Culture Collection (ATCC, Manassas, VA, USA). Cells were grown in Dulbecco’s modified Eagle’s medium (DMEM) supplemented with 10% fetal bovine serum (FBS), penicillin-streptomycin sulfate (100 units/mL and 100 µg/mL) at 37 °C in a humidified atmosphere of 5% CO_2_ incubator. Hs68 cells and HaCaT cells were seeded at a density of 1 × 10^5^/mL in a culture plate overnight. Thereafter, the cells were washed with phosphate buffered saline (PBS), and then exposed to UVB (15 mJ/cm^2^) using a UVP Crosslinker CL-1000M (Analytik Jena AG, Jena, Germany) as previous reports described [10]. Following that, incubated with or without various concentrations of EEB (25, 50, and 100 µg/mL) in DMEM.

### 4.3. Cell Viability Assay

Cell viability was determined using the MTT colorimetric assay. After UVB exposure, Hs68 cells were incubated in DMEM with different concentrations of EEB (25, 50, and 100 µg/mL) for 48 h. The cells were treated with MTT solution (5 mg/mL), followed by 2 h of incubation. And then, dimethyl sulfoxide (DMSO) was added to dissolve the formazan crystals. The absorbance was read at 540 nm using a microplate reader (Molecular Devices Inc., San Jose, CA, USA).

### 4.4. Analysis of MMP-1 and Pro-Collagen Type I Production

The production of MMP-1 (Cat.No.ab215083; Abcam, Cambridge, UK, Cat.No.E-EL-M0779; Elabscience Biotechnology Inc., Houston, TX, USA) and pro-collagen type I (Cat.No.MK101; TaKaRa Bio Inc., Shiga, Japan) in cell culture media and dorsal skin tissue-lysates was analyzed by using ELISA kits according to the manufacturer’s instructions. The absorbance was read at 450 nm using a microplate reader (Molecular Devices Inc., San Jose, CA, USA).

### 4.5. Analysis of Western Blots

The protein levels of Hs68 cells and dorsal skin tissues of HR-1 hairless mice were analyzed by Western blot according to our previous methods [10,49,50]. The proteins in Hs68 cells and dorsal skin tissues of HR-1 hairless mice were extracted using a PRO-PREP (Intron Biotechnology, Seoul, Korea) containing protease and phosphatase inhibitors. Then, the protein concentrations were estimated by Bradford’s assay. The proteins were separated by 8 to 15% sodium dodecyl sulfate-polyacrylamide gel electrophoresis (SDS-PAGE) gels and transferred onto polyvinylidene fluoride (PVDF) membrane. After the membrane was incubated overnight at 4 °C with primary antibodies at a concentration of 1:1000 in 2.5 to 5% skim milk, followed by 2 h incubation at room temperature with corresponding secondary antibodies at a concentration of 1:2000 in 2.5 to 5% skim milk. The blots were analyzed using an enhanced chemiluminescence (ECL) substrate and imaged by LAS-4000 luminescent image analyzer (FUJIFILM, Tokyo, Japan).

### 4.6. Analysis of Quantitative qRT-PCR

The mRNA levels of HaCaT keratinocytes were analyzed by qRT-PCR using the LightCycler 96 Instrument (Roche Molecular Systems Inc., Basel, Switzerland) as our previous report [10,49,50]. Total cellular RNA in HaCaT cells was extracted by using Easy Blue^®^ kits (Intron Biotechnology, Seoul, Korea), synthesis to cDNA using 0.5 mg/mL random oligonucleotide primers (Promega, Madison, WI, USA) and TOPscript^TM^ RTDryMIX (Enzynomics, Daejeon, Korea). PCR amplification was analyzed by using the incorporation of SYBR green (TaKaRa, Shiga, Japan) and specific primers. Primer sequences are listed in Table S1.

### 4.7. Measurement of Intracellular ROS Production

Hs68 cells were seeded at a density of 0.5 × 10^5^/mL in a culture plate overnight. After incubation, cells were replaced with fresh DMEM and stabilized for 48 h. The cells were exposed to UVB (15 mJ/cm^2^), treated with various concentrations of EEB in DMEM, and stained with 20 µM H_2_DCFDA in PBS for 30 min. The intracellular ROS productions were analyzed by fluorescence-activated cell sorting (FACS) cytometer (Cytomics FC 500, Beckman Coulter, CA, USA).

### 4.8. Animals

Five-week-old male HR-1 hairless mice were obtained from SLC Inc. (Shizuoka, Japan). The mice were housed in a temperature-controlled room (at 22 ± 1 °C and 40 to 60% humidity) with a 12/12 h light/dark cycle and permitted free access to food and water. The animal experimental protocols were approved by the Institutional Animal Care and Use of Laboratory Animals of Kyung Hee University Committee (Permit number: KHSASP-21-108).

### 4.9. Skin Photoaging Model

Animals were acclimated for 1 week before the start of the experiment. After then, thirty mice were divided randomly into five groups (*n* = 6/group) as follows: vehicle-treated control group without UVB exposure, UVB only-treated group, and UVB + EEB-treated groups (50, 100, and 200 mg/kg, p.o.). Each group was orally administered with vehicle or EEB 6 days per week. All groups, except the control group, were exposed to UVB three times per week using the UVP Crosslinker CL-1000M (Analytik Jena AG, Jena, Germany) for 8 weeks. The UVB concentration started at 60 mJ/cm^2^ (1st to 3rd weeks) and progressively increased to 120 mJ/cm^2^ (4th to 6th weeks), and 180 mJ/cm^2^ (7th to 8th weeks) in accordance with previous studies [10,49].

### 4.10. Analysis of Skin Wrinkle Formation

The dorsal skin of mice was replicated using the SILFLO kit (Monaderm, Monaco) for the measurement of skin wrinkle formation. The skin replicas were analyzed using a Visioline^®^ VL 650 (Courage & Khazaka Electronics GmbH, Cologne, Germany) for the following six indicators of wrinkles; the number of wrinkles, total length, mean length, wrinkle depth, mean depth, and max wrinkle depth.

### 4.11. Histological Analysis

The dorsal skin tissues of mice were obtained, fixed in 4% formaldehyde, and embedded in paraffin. The paraffin-embedded sections were stained with H&E and Masson’s Trichrome for histological analysis.

### 4.12. Measurement of Dorsal Thickness, Skin Hydration and Skin TEWL

At the end of the experiment, skin thickness, skin hydration, and TEWL in the dorsal skin of mice were measured by using a caliper (CD-AX/C, Mitutoyo Inc., Kanagawa, Japan) and GPSkin Barrier^®^ (Gpower Inc., Seoul, Korea). The values of skin hydration (range 0 to 100) and TEWL (range 0 to 80) were automatically calculated and expressed in arbitrary units (a.u.) and g/m^2^/h, respectively.

### 4.13. Analyze of Hepatoxicity and Renal Toxicity of EEB

The levels of GOT, GPT, BUN, and creatinine in HR-1 hairless mice plasma were analyzed using kits from T&P Bio (Gwangju, Korea). The plasma samples were obtained on sacrifice day, and were separated from the blood.

### 4.14. Statistical Analysis

The data are shown as the mean ± SD for in vitro and the mean ± SEM (*n* = 6) for in vivo. Statistically significant values were analyzed by one-way analysis of variance (ANOVA) and Dunnett’s post hoc test using GraphPad Prism software (GraphPad Software, Inc., San Diego, CA, USA). A value of *p* < 0.05 was considered significant. # *p* < 0.05 compared with the non-UVB-irradiated control group or the vehicle-treated control group, and * *p* < 0.05, ** *p* < 0.01, *** *p* < 0.001 compared with the UVB-irradiated group or the UVB only-treated group.

## 5. Conclusions

The present study revealed the protective effects of EEB and its underlying mechanisms in UVB-induced photoaging in vitro and in vivo. EEB prevented collagen breakdown by suppressing MMP-1 via the MAPK/AP-1 signaling pathway, increasing the expression of TIMPs, and enhancing collagen synthesis by stimulating the Smad signaling pathway. EEB reduced intracellular ROS levels and increased the Nrf2-mediated antioxidant system against oxidative stress. Moreover, EEB increased the mRNA expression of skin moisturizing factors, including filaggrin, involucrin, and loricrin, in UVB-irradiated HaCaT cells. Oral administration of EEB improved UVB-induced skin photoaging by inhibiting wrinkle formation, skin thickness, and collagen fiber destruction in the dorsal skin of hairless mice. EEB exerted its skin-hydrating effect by ameliorating the epidermal water content and TEWL. Taken together, EEB could protect the skin from aging and dryness by regulating MAPK/AP-1, Smad, and ROS-mediated Nrf2 pathways, and skin barrier function.

## Figures and Tables

**Table 1:** Retention time (Rt), precursor ion, monoisotopic mass, mass difference, and UV λmax of the identified peaks of EEB.

Compound	Rt (min)	Precursor Ion *(m/z)*	Monoisotopic Mass (M, AMU)	Mass Difference (mmu)	UV λ_max_ (nm)
**1**. Eckol	6.52	373.05449 [M+H]^+^	372.04813196	−1.47	230, 291
**2**. Phloroeckol	7.03	497.06954 [M+H]^+^	496.06417594	−2.46	232
**3**. 6,6′-bieckol	9.50	743.08752 [M+H]^+^	742.08061385	−0.92	294
**4**. 6,8′-bieckol	10.24	743.08724 [M+H]^+^	742.08061385	−1.19	292
**5**. 8,8′-bieckol	10.53	743.08790 [M+H]^+^	742.08061385	−0.54	291
**6**. Dieckol	19.55	743.08856 [M+H]^+^	742.08061385	+0.12	234, 291
**7**. Phlorofucofuroeckol A	23.07	603.07353 [M+H]^+^	602.06965524	−3.95	-

## Supplementary Materials

The following are available online at https://www.mdpi.com/article/10.3390/md19120693/s1, Table S1: Primer sequences, Figure S1: Effects of eckol and dieckol on cell viability and productions of matrix metalloproteinases-1 (MMP-1) and pro-collagen type I in UVB-irradiated Hs68 cells., Figure S2: Hepatotoxicity and renal toxicity of EEB in HR-1 hairless mice.
