# Supplementary material for: Eisenia bicyclis Extract Repairs UVB-Induced Skin Photoaging In Vitro and In Vivo: Photoprotective Effects
Source: Mar Drugs. 2021 Dec 3;19(12):693. doi: 10.3390/md19120693 (PMC8709268; doi:10.3390/md19120693)
Supplement: Supplementary file 1 [file marinedrugs-19-00693-s001.zip › marinedrugs-1483342-supplementary.pdf]

Supplementary Data

Table S1. Primer sequences

Type	Gene Description	Sequences (5′ → 3′)
Human	Filaggrin	F : AGTGCACTCAGGGGGCTCACA<br>R : CCGGCTTGGCCGTAATGTGT
	Involucrin	F : TTGGTCAGTGAAGCGATGAG<br>R : AGATCTGTCTGCAGGGCTGT
	Loricrin	F : GGCTGCATCTAGTTCTGCTGTTTA<br>R : CAAATTTATTGACTGAGGCACTGG
	Glyceraldehyde 3-phosphate dehydrogenase (GAPDH)	F : ATCAAGTGGGGCGAT GCTG<br>R : ACCCATGACGAACATGGGG

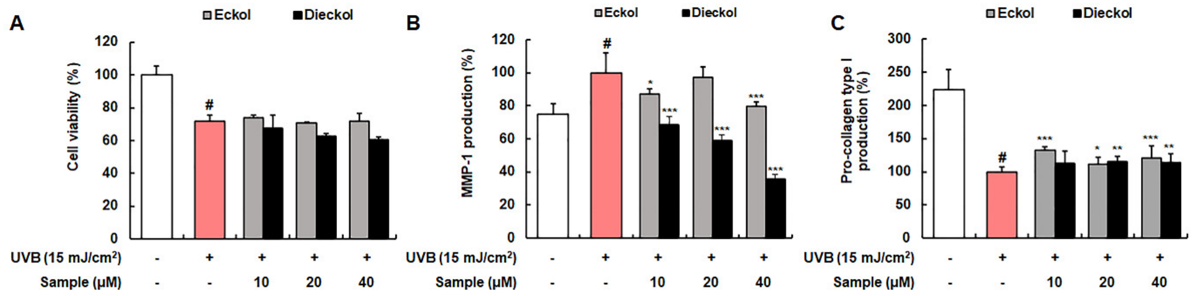

Figure S1. Effects of eckol and dieckol on cell viability and productions of matrix metalloproteinases-1 (MMP-1) and pro-collagen type I in UVB-irradiated Hs68 cells. Cells were irradiated by UVB and then treated with various concentrations of eckol and dieckol (10, 20, and 40 μM). **(A)** The cell viability was analyzed by using the 3-(4,5-dimethylthiazol-2-yl)-2,5-diphenyl tetrazolium bromide (MTT) assay. **(B, C)** The MMP-1 and pro-collagen type I levels in the cell culture media were analyzed by using ELISA kits. Values are represented as the mean ± standard deviation (SD). $^{\#}p < 0.05$ vs. the non-UVB-irradiated control group; $^{*}p < 0.05$, $^{**}p < 0.01$, and $^{***}p < 0.001$ vs. the UVB-irradiated group.

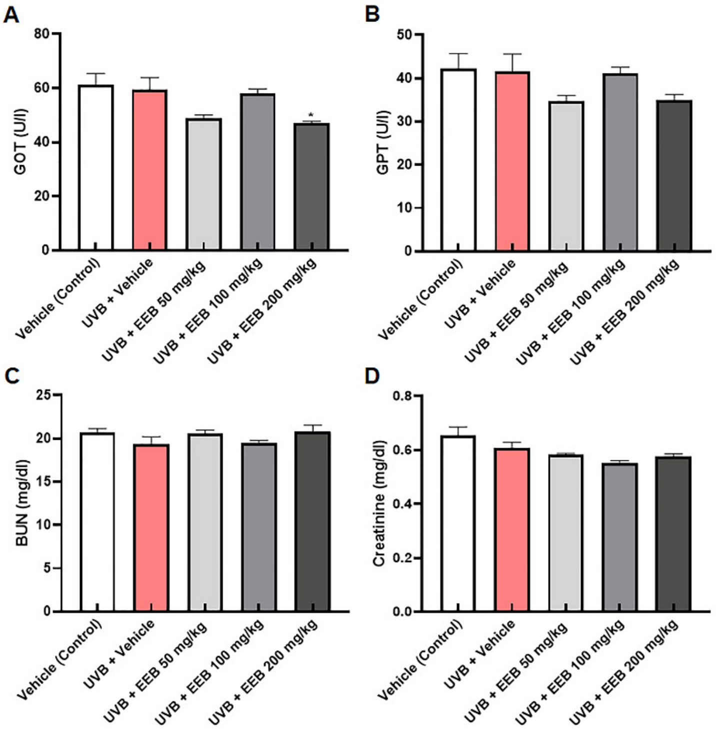

Figure S2. Hepatotoxicity and renal toxicity of EEB in HR-1 hairless mice. Biochemistry tests including **(A)** glutamic oxaloacetic transaminase (GOT, AST), **(B)** glutamic pyruvic transaminase (GPT, ALT), **(C)** blood urea nitrogen (BUN), and **(D)** creatinine in plasma of tested HR-1 hairless mice. Values are represented as the mean ± SEM (n = 6). $^{\#}p < 0.05$ vs. the vehicle-treated control group; $^{*}p < 0.05$ vs. the UVB only-treated group.
